# Prevalence of systemic inflammatory response syndrome (SIRS) in hospitalized children: a point prevalence study

**DOI:** 10.1186/1471-2431-9-25

**Published:** 2009-04-03

**Authors:** Jana Pavare, Ilze Grope, Dace Gardovska

**Affiliations:** 1 Riga Stradins University, Chair of Pediatrics, Riga, Latvia

## Abstract

**Background:**

In accordance with the 1st International pediatric sepsis consensus conference, where sepsis was defined as SIRS associated with suspected or proven infection, we have identified the need to assess the prevalence of SIRS and sepsis in children with abnormal temperatures hospitalized in The Children's Clinical University Hospital in Latvia.

**Methods:**

A descriptive prospective point prevalence study (using two time periods, each 24 h, randomly chosen) was conducted on all children (n = 943) treated in the hospital. All children with abnormal temperatures – fever or hypothermia (n = 92) – were included in the study. Questionnaires evaluating age-specific SIRS criteria were completed. The prevalence of SIRS was detected with 95% CI.

**Results:**

Out of a total of 943 patients treated in the hospital, 10% (n = 92) had abnormal temperatures. In all these cases the abnormal temperature was a fever; hypothermia was not established in any patient. Of the children with fever, 72% (n = 66) had SIRS. Of the SIRS patients, 8% (n = 5) developed sepsis, 5% (n = 3) severe sepsis and 2% (n = 1) septic shock. Seventy-six percent (n = 50) of the SIRS patients had fever in combination with respiratory rate >2 SD above normal for age; 50% (n = 33) had fever with abnormal leukocyte count; 15% (n = 10) had fever with tachycardia >2 SD above normal for age. Most of the SIRS patients (39%, n = 25) were aged 2–5 years. Twenty-one percent (n = 14) of the children with SIRS and 50% (n = 2) of those with severe sepsis and septic shock had an underlying disease. In no case was SIRS and sepsis recognized by doctors and the diagnoses were not recorded on the patients' cards.

**Conclusion:**

Our results would indicate a high risk for sepsis development in children with SIRS. Early SIRS diagnosis and awareness of risk of developing sepsis could change the medical approach to the patient in everyday clinical practice, eventually leading to early, goal-directed therapy for sepsis.

## Background

Sepsis has been recognized as a significant health problem among children and still remains one of the leading causes of morbidity and mortality in the child population [1-3]. In a population-based study in the United States, >42,000 cases of severe sepsis were reported in children per annum, with mortality 10.3% or > 4300 nationally [4]. Unfortunately, no data have been published about the frequency of sepsis in children in Latvia.

The International consensus conference on pediatric sepsis and organ dysfunction was held in February 2002 in San Antonio, where the criteria for adult systemic inflammatory responses syndrome (SIRS) were modified for pediatric use and definitions of sepsis, severe sepsis and septic shock for the pediatric population were revised [5]. Age-specific norms of vital signs and laboratory data were incorporated into the definitions of SIRS and sepsis in children [5,6]. Specific norms of vital signs and laboratory data were set for six clinically and physiologically meaningful age groups [5,6]. The major novelties in the definition of SIRS for children were a mandatory requirement for abnormality of temperature or leukocyte count for a diagnosis of SIRS in a pediatric patient [5]. The Institute for Healthcare Improvement (Cambridge, Massachusetts) has identified sepsis as an area of investigation. One deficiency that leads to suboptimal care of patients with severe sepsis is inconsistency in the early diagnosis of severe sepsis and septic shock [7,8].

In everyday clinical practice, one of the major challenges is still to recognize sepsis when it starts. In its early stages, sepsis is often mistaken for a milder infection or a condition resulting from some other therapeutic or surgical cause. Clinical practice worldwide, and also in Latvia, demonstrates that some pediatricians associate the term sepsis with septic shock: only when the blood pressure drops, the patient no longer responds to intravenous fluids, or respiratory failure develops requiring mechanical ventilator support, do pediatricians agree that the patient has septic shock; other doctors agree that the patient is septic if a positive blood culture is confirmed [9]. Erroneously, a patient is not considered to be septic on the basis of pneumonia, high fever and elevated blood count or tachycardia.

Rapid recognition and diagnosis of sepsis is critical, and early goal-directed therapy can be very effective [8,10,11]. Relatively simple strategies have been used in the developing world to identify sepsis, and well-timed appropriate treatments, which include empirical antibiotics used in newborns and infants [12,13] and early aggressive fluid resuscitation [14], have met with considerable success.

In view of the current definition of sepsis in children and infants where SIRS plus infection equals sepsis, it is crucially important to recognize systemic inflammatory response syndrome (SIRS) patients in everyday practice and to appreciate the importance of multifactorial management of the potential sepsis sufferer.

The aim of this study was to assess the prevalence of SIRS and sepsis among hospitalized children in different age groups with abnormal temperatures in the Children's Clinical University Hospital.

## Methods

### Study population and inclusion criteria

**Figure 1:**
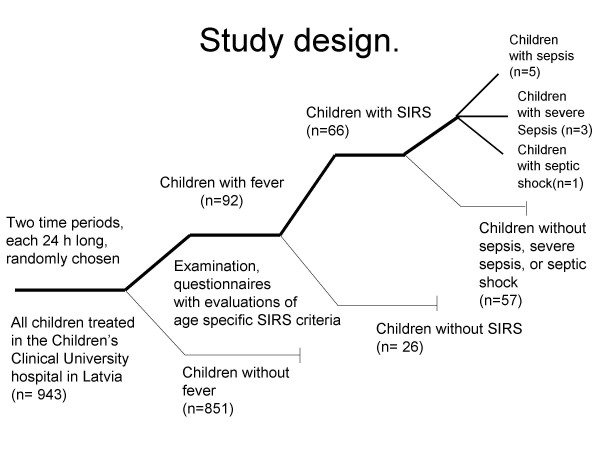
**Study design**.

This descriptive prospective point prevalence study was performed in The Children's Clinical University Hospital in Latvia, which is a tertiary level hospital. Two time periods, each 24 h, were randomly chosen in January and February 2007. All patients (n = 943) treated in the hospital during these two time periods (n = 456 and n = 487 respectively) were screened, and all children with abnormal temperatures (n = 92) were included in the study (Figure 1). Children with elevated or decreased leukocyte count for age were also included in the study irrespective of their temperature. For all children with abnormal temperatures or leukocyte counts, a questionnaire was completed recording heart rate, respiratory rate, core temperature and leukocyte count.

The patients were divided into six age groups as proposed by the International consensus conference on pediatric sepsis: newborn, neonate, infant, toddler and preschool, school age, adolescent and young adult [5]. We evaluated the age-specific vital signs and laboratory variables for each child. SIRS was confirmed in accordance with the definition of systemic inflammatory response syndrome [5]: at least two of the following four criteria must be met, one of which must be abnormal temperature or leukocyte count:-

• Core temperature >38.5 or < 36.0°C.

• Tachycardia, defined as a mean heart rate >2 SD above normal for age; or for children <1 yr old, bradycardia, defined as a mean heart rate <10th percentile for age.

• Mean respiratory rate >2 SD above normal for age.

• Leukocyte count elevated or depressed for age or >10% immature neutrophils.

The prevalence of SIRS among hospitalized children was detected with 95% confidence intervals. The prevalence of sepsis among children with SIRS was also determined.

We assessed the patients according to age-specific vital signs and laboratory values to determine the criteria for diagnosis of SIRS and to gain clinical and demographical data about SIRS and sepsis patients.

The participation of patients was voluntary. Before the study, each child's parents signed a consent form approved by the Central Medical Ethics Committee.

During the study period we followed the subject's disease process, analyzed the outcome of the disease and studied the final diagnosis, but we did not interfere in the treatment.

### Data analysis

The study data were processed using EPI INFO 2000 and SPSS. For the analysis of patients' data, descriptive statistics were used. For the analysis of patient, data descriptive statistics were used. For continuous variables, normality was checked. An appropriate non – parametric test was chosen, since the data were not distributed normally. Comparisons of continuous variables between groups were applied using the Mann – Whitney and Wilcoxon tests. Results were presented as numbers (n), percent (%), means and minimum – maximum (min-max). A p value < 0.05 was considered statistically significant.

## Results

### SIRS and sepsis prevalence

A total of 943 patients were treated in the hospital, 456 in the first time period and 487 in the second. Abnormal temperature was prevalent in 92 patients (10%). All these children had fevers. No patient had hypothermia. Seventy-two percent (n = 66) of the children with fever had SIRS, 8% (n = 5) of the SIRS patients in our study developed sepsis, 5% (n = 3) severe sepsis and 2% (n = 1) septic shock. The prevalence of SIRS among all the hospitalized children was 0.07 ± 0.016, but among the children with fever it was 0.72 ± 0.092.

### Confirmation of SIRS diagnosis

A diagnosis of SIRS, in accordance with the definition of systemic inflammatory response syndrome in children, was mostly confirmed by the combination of fever with respiratory rate >2 SD above normal for age (76%, n = 50). Fever with abnormal leukocyte count was present in 50% (n = 33), and fever with tachycardia >2 SD above normal for age in 15% (n = 10). When children with leukocyte counts elevated or depressed for age but without temperature abnormalities were also included, only one additional child met the SIRS criteria.

### Age groups, wards where children were treated and underlying diseases (premorbid conditions)

Thirty-nine percent (n = 25) of the SIRS patients were in the 2–5 year age group, 25% (n = 17) in the 1 month-1 year group, 21% (n = 14) in the 13 to <18 year group and 15% (n = 10) in the 6–12 year group. In neither time period did we identify any patients in the newborn and neonate age groups. Fifty-six percent (n = 37) of the SIRS patients were treated in infectious diseases departments, 24% (n = 16) in general pediatrics, 12% (n = 8) in surgical and 8% (n = 5) in ICU. Twenty-one percent (n = 14) of the children with SIRS and 50% (n = 2) of children with severe sepsis and septic shock had an underlying disease.

### Sepsis diagnosis

Sepsis, according to definition of the International consensus conference on pediatrics, was confirmed in 60% of cases by the combination of SIRS with suspected infection and in 40% of cases by the combination of SIRS with proven infection. Only 40% of the sepsis patients were treated in the ICU.

### Clinical and demographical data of SIRS and sepsis patients

No gender difference was detected among the SIRS patients (boy/girl ratio 36/30 in the SIRS group). Median length of hospitalization was 7 days for SIRS patients (mean 11.3 days, range 2–62 days) and 9 days (mean16.2 days, range 4–44 days) for sepsis patients. Antibacterial therapy was used for 33% of SIRS patients (n = 22) and 100% of sepsis patients. In median antibacterial therapy was started on the 1^st ^day of hospitalization (on average 2^nd^, earliest on the 1st day, latest on the 13^th ^day) in SIRS patients and on the 1^st ^day for all sepsis patients. The median duration of antibacterial therapy was 5 days in SIRS patients (mean 5.6 days, minimum 0 days, maximum 15 days) and 8 days in sepsis patients (mean 8.8 days, minimum 4 days, maximum 15 days) (Table 1). Fatal cases were not established in this study. None of the SIRS and sepsis cases were recognized by doctors and recorded on the patients' cards.

**Table 1:** Clinical and demographical data of SIRS and sepsis patients.

	SIRS (n = 66)	Sepsis, severe sepsis, and septic shock (n = 9)	*P *value
Gender boys/girls (number)	36/30	5/4	
Duration of hospitalization/days (median, minimum – maximum)	7 (2–62)	16.2 (4–44)	NS
Antibacterial therapy	33% (n = 22)	100% (n = 9)	
Day of initialization of antibacterial therapy (median, minimum – maximum)	1(1–13)	1 (1-1)	NS
Duration of antibacterial therapy/days (median, minimum – maximum)	5 (0–15)	8.8 (4–15)	< 0.05

SIRS, systemic inflammatory response syndrome. NS, not significant.

## Discussion

Several studies on adults and children have established the prevalence of systemic inflammatory response syndrome (SIRS) and sepsis. We used the National Library of Medicine (National Institutes of Health) database for a literature search and found a relatively low number of publications on this subject. We could find no publications describing the prevalence of SIRS among patients with abnormal temperatures in children population.

In our study, the prevalence of SIRS among hospitalized children with fever was 72%. Our literature review revealed one study reporting a 68% prevalence of SIRS in pediatric intensive care units [15]; in the adult population, 68% of patients admitted to three intensive care units and three general wards met at least two criteria for SIRS [16]. In Japan, the prevalence of SIRS reached 84% among all adult ICU patients [17]. SIRS affected one third of all hospitalized patients in the study by Brun-Buisson [18]; it developed in 59% of critically ill obstetric patients [19] and 82% of admissions to a university hospital in Canada [20]. Among hospitalized adult medical patients with new onset of fever in the department of internal medicine, 95% had SIRS [21]. These data are not fully comparable with our data because different patient populations were studied.

Eight percent of the SIRS patients in our study developed sepsis, 5% severe sepsis and 2% septic shock. Our data are consistent with other publications showing that severe sepsis and septic shock occurred in 2–3% of ward patients [18], and 8% of patients with SIRS progressed to severe sepsis [17]. However, some studies have reported higher rates of sepsis: among adult patients with SIRS at three intensive care units and three general wards, 26% developed sepsis, 18% severe sepsis and 4% septic shock [16]. In another study on children, 23% of intensive care unit patients had sepsis, 4% had severe sepsis and 2% had septic shock [20]. The variances between the rates of sepsis occurrence could be explained by the different patient populations in the references. Our data are comparable to the studies that looked at general hospital wards but they differ from studies that looked at the intensive care patient population. We can note that rates for sepsis development are higher among intensive care unit patients.

The diagnosis of SIRS in our study was based on a combination of abnormal temperature with respiratory rate >2 SD above normal for age in 76% of cases, a combination of abnormal temperature with abnormal leukocyte count in 50%, and abnormal temperature with tachycardia >2 SD above normal for age in 15%. Carvalho et al. [15] in a study conducted in a pediatric intensive care unit found that increased heart rate >2 SD above normal value for age occurred in 85% of SIRS patients, abnormal leukocyte count in 77%, and increased respiratory frequency >2 SD above normal value of age in 47.5%.

We found that most (39%) of the SIRS patients were in the 2–5 year age group with a median age of 30 months, which is close to the median age of SIRS patients (24 months) found in another study [15]. In the United States in 1995, the incidence of severe sepsis was highest in infants [4]; in our study, the infant group was second in order of frequency of SIRS [4]. Twenty-one percent of children with SIRS in our study had an underlying disease; in contrast, Carvalho et al. [15] noted that 64% of children with SIRS had no comorbidity. In our study, 50% of the patients who developed severe sepsis or septic shock had an underlying disease. Watson et al. [4] in the United States studied children with severe sepsis using 1995 hospital discharge documentation and found that half the cases (49%) had underlying disease. This is generally consistent with the conclusions from a study conducted on an adult population [18], where the major determinant of prognosis of septic syndromes was related to underlying diseases.

We found no gender difference among SIRS patients, in agreement with Carvalho et al., but in another study the incidence of severe sepsis was 15% higher in boys than in girls [4].

Including a time period longer than 48 hours and preferably in different seasons should be necessary to elucidate the impact of seasonal infections on SIRS and sepsis frequency in hospitalized children.

We would like to point out that laboratory tests are as important as physiological parameters for the early diagnosis of sepsis. During the last decade different biochemical markers of sepsis – C reactive protein (CRP), procalcitonin (PCT), inflammatory cytokines and acute phase reactants have been successfully studied and used in clinical practice in determining the onset of sepsis and quantifying the response to therapy.

## Conclusion

There was a high prevalence of SIRS (72%) among hospitalized children with abnormal temperature in The Children's Clinical University Hospital in Latvia, the risk of developing sepsis was considerable: 8% of SIRS patients developed sepsis, 5% severe sepsis and 2% septic shock. Isolated abnormal leukocyte count without temperature abnormality did not meet the criteria for prompt diagnosis of SIRS. Timely recognition and understanding of the role of SIRS in the development of sepsis will contribute to early diagnosis of sepsis in everyday practice. The specially modified SIRS criteria for children and revised definitions of severe sepsis and septic shock for the pediatric population will improve stepwise and more targeted diagnosis of sepsis. Once SIRS is identified, it is crucially important to keep the patient under observation so that rapid and appropriate treatment can be initiated.

## Competing interests

The authors declare that they have no competing interests.

## Authors' contributions

JP planned the study, wrote the protocol, collected and analyzed data, wrote the report. IG was responsible for study planning, collection and analyzing of data, she was involved in revision of manuscript and in practical clinical aspects. DG was involved in study planning, in revising the manuscript.

## Pre-publication history

The pre-publication history for this paper can be accessed here:


